# Critical appraisal of arguments for the delayed-start design proposed as alternative to the parallel-group randomized clinical trial design in the field of rare disease

**DOI:** 10.1186/s13023-017-0692-3

**Published:** 2017-08-17

**Authors:** Loukia M. Spineli, Eva Jenz, Anika Großhennig, Armin Koch

**Affiliations:** Institute for Biostatistics, Hannover Medical School, Carl-Neuberg-Straße 1, 30625 Hannover, Germany

**Keywords:** Delayed-start, Randomized trial, Orphan drug, Rare disease, Sample size, Treatment effect

## Abstract

**Background:**

A number of papers have proposed or evaluated the delayed-start design as an alternative to the standard two-arm parallel group randomized clinical trial (RCT) design in the field of rare disease. However the discussion is felt to lack a sufficient degree of consideration devoted to the true virtues of the delayed start design and the implications either in terms of required sample-size, overall information, or interpretation of the estimate in the context of small populations.

**Objectives:**

To evaluate whether there are real advantages of the delayed-start design particularly in terms of overall efficacy and sample size requirements as a proposed alternative to the standard parallel group RCT in the field of rare disease.

**Methods:**

We used a real-life example to compare the delayed-start design with the standard RCT in terms of sample size requirements. Then, based on three scenarios regarding the development of the treatment effect over time, the advantages, limitations and potential costs of the delayed-start design are discussed.

**Results:**

We clarify that delayed-start design is not suitable for drugs that establish an immediate treatment effect, but for drugs with effects developing over time, instead. In addition, the sample size will always increase as an implication for a reduced time on placebo resulting in a decreased treatment effect.

**Conclusions:**

A number of papers have repeated well-known arguments to justify the delayed-start design as appropriate alternative to the standard parallel group RCT in the field of rare disease and do not discuss the specific needs of research methodology in this field. The main point is that a limited time on placebo will result in an underestimated treatment effect and, in consequence, in larger sample size requirements compared to those expected under a standard parallel-group design. This also impacts on benefit-risk assessment.

## Background

Individual randomized controlled trials (RCTs) are placed in the top of the hierarchy of quality evidence after systematic reviews of good quality RCTs and thus, they have been advocated as the gold standard for the comparison and evaluation of the efficacy of different medical interventions [1, 2]. Orphan legislation requests that drugs for rare diseases are tested and licensed according to the same rules as established for common diseases. Deviations from such rules should be prospectively justified in the protocol and further elaborated in the study report [3]. The Guideline on Clinical Trials in Small Populations states that the majority of orphan drugs and pediatric indications submitted for regulatory approval are based on RCTs conducted according to established regulations and guidance and this has been reinforced in a recent report from the German Institute for Quality and Efficiency in Medal Health [3, 4].

At the same time, published literature underlines the difficulties and challenges in conducting standard parallel group RCTs in rare diseases. Main reasons are sample-size restrictions due to low prevalence that defines these diseases and the need to conduct multiregional clinical trials [5–7]. Specifically, multiregional clinical trials may bear the risk of increased heterogeneity of the patient population due to genetic or environmental factors whereas small regional trials have a high risk of failure, or being stopped without sufficient recruitment in a reasonable time-frame. Consequently, in most instances there are no alternatives to the conduct of multi-regional clinical trials to arrive at a sufficient sample-size.

Many rare diseases are enzyme deficiency diseases diagnosed early in childhood. The general reluctance to include children into RCTs is an additional barrier to the recruitment. In order to overcome these challenges, other designs beyond the standard parallel-group RCT have been proposed to ease recruitment for the study of interventions in rare disease. Such design examples constitute the cross-over design, multiple n-of-1 design, Bayesian adaptive designs and enrichment designs, such as randomized withdrawal design.

Several articles have been published that discuss the main characteristics, advantages and limitations of these designs (for instance, [8, 9]) in order to assist the researcher in the selection of the most appropriate design for a specific clinical situation in the field of rare disease. The choice of the most appropriate design is not an easy task and overall feasibility, acceptability and potential biases to the study’s internal and external validity require consideration [10].

A further investigation of the literature revealed that actually in many instances recommendations in papers had low validity when critically challenged against the fact that at least in rare diseases the main interest is to arrive at a trial design that requires lower sample-size and has higher efficiency as compared to the standard parallel group design [8, 9, 11]. The implications for using alternative designs in terms of sample-size, bias, or overall information have not been properly explained. Furthermore, the authors have not elaborated on why claimed advantages are of specific importance in rare diseases; for instance, why limited placebo exposure is more important in rare than in common diseases [8, 9, 11]. Unfortunately, the authors, in an effort to suggest a design as advantageous alternative in the field of rare disease, tend to use the same argumentation as in common diseases and do not discuss potential implications for usage in rare disease.

An example of such literature is a recent paper on experimental design recommendations for small populations which did raise our interest [8]. The discussion did not provide enough detail to really decide about the advantages and disadvantages of the designs proposed in terms of overall efficacy and sample size requirements in the context of rare disease. The authors developed an algorithm to assist the choice of an appropriate design in the field of rare disease. However, the decision criteria of this algorithm are not at all specific for the situation of a rare disease, but apply to common disease in the same way and the discussion of the potential implications is rather limited.

Among the designs discussed in [8], we chose the delayed-start design for a more elaborated discussion about its potential advantages and disadvantages. The delayed-start design was initially discussed for the investigation of pediatric diseases in an attempt to reduce the time on placebo, or on an inferior treatment, and to limit potential disadvantages of a placebo treatment. The design found renewed interest in Alzheimer’s disease in an attempt to find a clinical definition for disease modification [12–14]. Claimed advantages of the delayed-start design thus include (i) to limiting duration of placebo treatment, (ii) to allowing all patients to receive the test treatment and (iii) to conclude, nevertheless, also on the disease-modification effects of the tested treatment.

In this article, we aim to evaluate advantages of the delayed-start design regarding overall efficacy and sample size requirements to justify application in the specific situation of rare diseases.

## Methods

### Sample size requirements using a real-life example

**Fig. 1:**
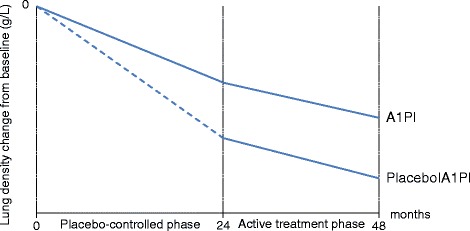
Annual rate of lung density change from baseline to 24 and 48 months in the RAPID trial [11]. At baseline (0 months), patients are randomly assigned to receive either A1Pl or placebo (placebo-controlled phase) and they are followed for 24 months. Then, patients in the placebo group switch to the investigational treatment for another 24 months (active treatment phase)

To investigate the sample size requirements under the delayed-start design compared to those under the standard RCT, we considered the RAPID (**R**andomised, placebo-controlled trial of augmentation therapy in **A**lpha-1 **P**roteinase **I**nhibitor **D**eficiency) study as a real-life example [15]. RAPID was a multicentre, double-blind, randomised, parallel-group, placebo-controlled trial of A1PI treatment in adult non-smoker patients with α1 antitrypsin deficiency. Primary endpoint of the study was an assessment of the CT lung density at total lung capacity and of the functional residual capacity, combined and separately, at 0, 3, 12, 21, and 24 months in a double-blind manner and then at 36 and 48 months in an open-label extension for both treatment groups. Therefore, the RAPID study can be conceived as a delayed-start design, where the first 2 years the patients were randomized to receive either A1PI or placebo, followed by switching patients in the placebo arm to A1PI for another 2 years [15, 16]. Positive difference in the annual rate of lung density change indicates superiority of A1PI treatment (Fig. 1).

We aim to investigate the scenario where trialists would have considered the two-year placebo to be too long and they would have wished to plan the study as a delayed-start design, where A1PI is delayed by 12 months in the placebo group. In the delayed-start group, we will assume a placebo annual rate of lung density change from baseline to 1 year followed by the 12 month effect of A1PI in the second year and we will use the annual rate of lung density change from baseline to 2 years for the early-start group [15]. With this information, we will calculate the sample-size and compare to the actual sample-size needs with observations after 2 years in the standard RCT (where patients would have been randomly assigned either to A1PI or placebo and would have remained in the assigned groups until termination of the trial).

We used the published results on annual rate of lung density change from baseline to 24 months which is equal to −2.19 g/L/year (SE 0.25) for the placebo group and −1.45 g/L/year (SE 0.23) for A1PI. In order to calculate the sample size requirements for delaying the treatment by 12 months in the placebo group, we assumed that the annual rate of lung density in the delayed-phase equals half the annual rate of lung density of the 24-month delayed-phase (i.e. -1.09 g/L/year), whereas we assumed that the annual rate of lung density in the open-label extension equals half the annual rate of lung density of the 24-month open-label extension (i.e. -0.65 g/L/year). Subsequently, the standard error for the 12-month delayed-phase was assumed to be half the standard error for the 24-month delayed-phase (i.e. 0.125 g/L/year), and similarly for the standard error for the 12-month open-label extension. Since there was no available information in either group on the standard error of the annual rate of lung density change from 24 to 48 months, we made the strong assumption that the standard error from the delayed-phase remained stable in the open-label extension.

To obtain the required sample sizes, we applied the two-group t-test of equal means and we chose a 5% significance level and 80% power. We used the nQuery Advisor® 7.0.

## Results

### Description of the delayed start design

**Fig. 2:**
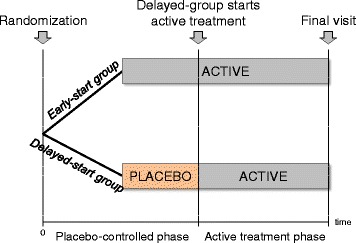
Graphical display of the delayed-start design. At baseline (time 0), patients are randomly assigned to receive either placebo (delayed-start group) or the investigational treatment (early-start group) and they are followed over an extended period of time. Then, patients in the placebo group switch to the investigational treatment until the end of the trial (active treatment phase)

The delayed start design is illustrated in Fig. 2. At baseline, patients are randomly assigned to receive either placebo (delayed-start group) or the investigational treatment (early-start group) for a certain period of time. Subsequently, the patients in the placebo-group switch to active treatment and they are followed for an extended period of time.

The data obtained at the end of the placebo-controlled phase allow inferences of causality, namely, any differences in the symptoms of the compared groups are due to genuine therapeutic potency of the investigational treatment on the symptoms rather than confounding or other biases. Nevertheless, the data obtained at the end of trial serve to investigate the disease-modifying effects of the investigational treatment.

### Investigating the sample size requirements using a real-life example

Delaying assignment to A1PI by 12 months leads to a smaller annual rate of lung density change from baseline to 24 months under the delayed-start design and the sample size required to achieve this change is tremendously large (Table 1). Contrariwise, a standard RCT can achieve larger effect in the 2-year time-frame with much smaller sample size requirements.

**Table 1:** Calculated sample size requirements

Parameters	Double-blind^a^	Open-label extension^b^
A1P mean (SE)	−1.45 (0.23)	−1.45 (0.23)
Placebo mean (SE)	−2.19 (0.25)	−1.74 (0.25)
Difference	0.74	0.29
Common SD	2.25	2.25
Effect size	0.32	0.13
N per group	*147*	*946*

*SE* standard error, *SD* standard deviation, *N* number of patients

^a^Annual rate of lung density change from baseline to 24 months (A1PI versus placebo)

^b^Annual rate of lung density change from baseline to 24 months (A1PI versus delayed-start A1PI)

In the next section we illustrate schematically the sample size and treatment effect considerations that have been demonstrated with the real-life example.

## Examples

### Suitability of the delayed start design

**Fig. 3:**
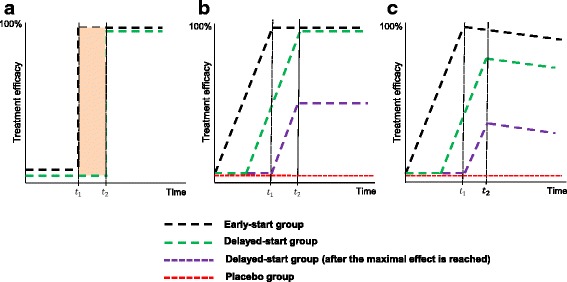
Treatment-effect development over time under the delayed-start design. The delayed-start design (early-start group versus delayed-start group) is compared with the standard parallel design (early-start group versus placebo group) in terms of **a** immediate treatment effects, **b** developing treatment effects reaching complete efficacy and **c** developing treatment effects with complete efficacy without reaching complete efficacy

Figure 3 schematically illustrates three different scenarios of treatment-effect development over time under the delayed-start design in order to understand the impact of how the treatment effect develops over time. Time is depicted on x-axis, whereas the treatment efficacy is presented as percentage on y-axis. The early-start group (i.e. the group directly assigned and maintained to the active treatment until trial completion) and the delayed-start group (i.e. the group initially assigned to placebo and then switched to the active treatment at some point in time) are defined by the black and green dotted line, respectively.

In Fig. 3a, a few time units after taking the active treatment (for instance, aspirin), an immediate effect is observed in the early-start group and achieves 100% of its efficacy at *t*_1_. The delayed-start group responds also immediately, but at a later timepoint *t*_2_ (*t*_2_ > *t*_1_) reaching also 100% of efficacy. Inferences can be made only on the immediate effect of these treatment groups within the time frame as indicated by the parallel vertical lines (i.e., between *t*_1_ and *t*_2_); the efficacy of the early-start group compared to that of the delayed-start group remains constant.

A careful investigation of Fig. 3a reveals that the delayed-start design is not suitable for drugs that establish an immediate treatment effect. If the treatment effect is investigated too early, it would be zero but then would be observed for the very short period that is required to establish the immediate effect, followed by zero effect, thereafter. As outlined above, the advantage of the design to limit duration on placebo is only becoming effective if the treatment effect is developing slowly over time. As a result, assumptions about the development of the treatment effect over time are implicit to this design approach.

### Treatment-effect development over time

In Fig. 3b, the treatment-effect of the early-start group starts immediately to develop and it reaches 100% of its efficacy at *t*_1_. The treatment-effect of the delayed-start group starts developing before early-start group reaches 100% of its efficacy and it arrives also at 100% efficacy later at *t*_2_. Inferences can be made only on the symptomatic effect of these treatment groups within the time frame as indicated by the parallel lines. Now consider the case of a parallel RCT instead of a delayed-start design; the active treatment (black dotted line) develops immediately and reaches 100% efficacy at *t*_1_, whereas the placebo group (red dotted line) has not responded yet. At *t*_1_ the treatment effect is smaller under delayed-start design than the standard parallel-group RCT, and as a result, the former fails to achieve proof of efficacy. The treatment-effect increases but remains smaller under the delayed-start design. The treatment-effect development has a different impact on the sample size of these two designs; a parallel RCT has smaller sample size requirements than a delayed-start design to capture a larger treatment effect than the latter.

In the special case, where the treatment-effect in the delayed-start group starts developing right after the early-start group reaches 100% of its efficacy (purple dotted line), the treatment-effect under the delayed-start design equals the treatment-effect under the parallel RCT at *t*_1_, but reduces at *t*_2_ without reaching 100% efficacy, though.

Similarly, in Fig. 3c, the treatment-effect of the early-start group develops immediately and it reaches 100% of its efficacy at *t*_1_. However, when progression ensues, the early-start group starts losing its efficacy in a continuum and the subsequent treatment-effect development plummets. The treatment-effect of the delayed-start group starts developing before early-start group reaches 100% of its efficacy but it does not arrives at 100% efficacy later at *t*_2_. After *t*_2_, a distance arises between the curve representing the treatment response for the patients in the early-start group and the curve representing the treatment response for the patients in the delayed-start group. Such a distance may be either constant or decreasing over time indicating alleged disease-modification effects [12].

When considering the special case, where the treatment-effect in the delayed-start group starts developing right after the maximal effect is reached in the early-start group (purple dotted line), the treatment-effect under the delayed-start design equals the treatment-effect under the parallel RCT at *t*_1_. However, at *t*_2_ the treatment-effect of the delayed-start group does not reach 100% efficacy and it starts plummeting afterwards.

## Discussion

In this article, we discussed the delayed-start design where we used pictorial examples and real-life data from the RAPID study to demonstrate that the implications of conducting a trial with delayed-start design include underestimation of true treatment effect and, consequently, larger sample size requirements compared to the standard RCT. Moreover, in the pictorial examples we illustrated that by knowing the exact time of effect in order to switch patients in the placebo-group to the active treatment, the treatment-effect (and by extension the sample size requirements) under the delayed-start design equals the treatment-effect under the standard RCT. However, the standard RCT still prevails over the delayed start design in terms of treatment-effect (and minimum sample size requirements) in the subsequent assessment time-point.

The acceptability of a placebo control arm is often seen as a conflict between the scientific standards and the ethical requirements of the need to treat patients, but is based on the assumption that the experimental treatment is better than no treatment, which is actually the objective of the trial. The ethical acceptability of the placebo is mainly a matter of design, trial duration, disease severity, and availability of therapeutic alternatives rather than the prevalence of the condition under investigation (i.e. common versus rare disease). There is a misconception that patients randomized to the placebo arm are left untreated. On the contrary, outside the direct comparison of the experimental and the control treatment, best standard of care should (and must) be exercised in both treatment groups to arrive at a valid comparison of treatment arms.

The advantage of placebo-controlled trials is the ability to distinguish between the adverse-events due to the treatment per se and those due to the studied condition, or co-medication. Again, the acceptability of the placebo control matters for all diseases irrespective of frequency and age populations (i.e. children, adults and elderly).

It has been reported that the limited time to placebo arm and the administration of the experimental treatment to all participants are particular features of this design that make it attractive to the research in rare disease, leastwise at first sight [8]. Moreover, this design can be attractive also to pediatric trials, where parents may be reluctant to enroll their child in a trial where he or she may be assigned from the beginning of the trial to placebo rather than to the experimental intervention for the whole duration of the trial [7]. Considering the enumeration of the advantages of this design within the field of common and rare disease, it is evident that there is nothing new to be added for the rare diseases other than what has been claimed for the common diseases. Additionally, the reputed feasibility of such a design in pediatric trials as more appropriate than a parallel RCT raises the following question; why limitation of placebo exposure in pediatric trials is more essential in rare than common diseases? Furthermore, none of the authors who have applauded this design as proper in the field of rare disease has ever mentioned the increased sample size requirements of this design compared to the standard parallel-group RCT. The former may unnecessarily expose some patients to placebo or to possible side-effects of the experimental treatment as well as waste time and precious research sources.

Furthermore, the implications of the delayed administration of the active agent on the risk-benefit assessment deserve discussion. Risk-benefit assessment requires a precise estimation of the benefit in order to balance against risk. A delayed-start design can be conducted to substantiate formal proof of efficacy (i.e. the drug is different from placebo) but for obvious reasons the true treatment effect will always be underestimated (Fig. 3c) merely due to the delayed initiation of the active treatment in the control group and the diminishing difference between treatments. As a result, the estimated treatment effect, when different from zero can inform whether the experimental treatment is superior to placebo, but for a benefit-risk evaluation only a lower boundary for the true treatment effect is available in the context of a delayed-start design.

Treatment-effects for diseases that progress slowly, like Alzheimer’s disease, Parkinson’s disease, rheumatoid arthritis, and chronic obstructive pulmonary disease can be modelled under a delayed-start design [12]. Alike Fig. 3b, the immediate implications by using a delayed-start design in order to evaluate the treatment-effects of a slowly but constant progressive disease is a much smaller treatment-effect than under a parallel RCT. As a result, the sample size requirements under the delayed-start design are increased. In rare diseases, where recruitment is constrained primarily due to inherent patient unavailability and heterogeneous set of conditions, it is impossible to recruit a large sample of patients or require prolonged trial duration and provident funding, posing an additional barrier to the execution of trials in rare diseases [7].

Zhang and colleagues (2011) also reported on the larger patient requirements and the longer duration of this design relative to current long-term randomized, double-blinded, placebo-controlled, parallel design trials of disease-modifying agents in Alzheimer disease [17]. Despite the shortcoming of larger sample size requirement, the authors applauded the delayed-start design for its alleged ability to detect a disease-modifying effect that constitutes the greatest advantage of this design. The question is whether a disease-modification effect is actually an existing reality or simply a theoretical consideration without a proof. It is further noted that a drug with slow onset may be identified as disease modifying in a trial with a delayed start design despite the fact that it is leading to symptomatic effects, only.

So far, attempts to identify disease-modifying agents have been proven unsuccessful in the field of common neurodegenerative diseases, such as Parkinson’s disease and dementia of Alzheimer type [18, 19]. Researchers attribute this deficiency to the lack of deep knowledge on the etiology and pathogenesis of most of those conditions. An agent achieves to establish disease-modifying effects when it delays the underlying pathological or pathophysiological disease processes and simultaneously, it improves the clinical signs and symptoms [18]. Improvement of clinical signs and symptoms alone is sufficient to claim a symptomatic effect.

## Conclusions

Despite the advantages that have been claimed for the delayed-start design in the context of rare diseases, there is no additional utility of the design recommendations in the field of rare disease beyond those seen in common diseases. Subsequently, we initiated a more specific discussion on the downsides of the delayed-start design: limited time in the placebo group will result in a smaller treatment effect and in consequence to larger sample size requirements compared to those expected under a standard RCT. Underestimation of the true treatment effect also hampers benefit/risk assessment. As in the end, all patients will be on the experimental treatment late negative consequences of drug treatment are more difficult to detect. We feel that there is utility in the delayed start design, but particularly under sample-size restrictions advantages and disadvantages deserve a thorough discussion before applying the design in the field of rare disease.

## Abbreviations

A1PI: Alpha-1 Proteinase Inhibitor
RAPID: Randomized, placebo-controlled trial of augmentation therapy in Alpha-1 Proteinase Inhibitor Deficiency
RCTs: randomized controlled trials
